# The monkeypox diagnosis, treatments and prevention: A review

**DOI:** 10.3389/fcimb.2022.1088471

**Published:** 2023-02-06

**Authors:** Saadullah Khattak, Mohd Ahmar Rauf, Yasir Ali, Muhammad Tufail Yousaf, Zhihui Liu, Dong-Dong Wu, Xin-Ying Ji

**Affiliations:** ^1^ Henan International Joint Laboratory for Nuclear Protein Regulation, School of Basic Medical Sciences, Henan University, Kaifeng, Henan, China; ^2^ School of Pharmaceutical Sciences, Wayne State University, Detroit, MI, United States; ^3^ National Center for Bioinformatics, Quaid-e-Azam University, Islamabad, Pakistan; ^4^ Institute of Microbiology, Faculty of Veterinary and Animal Sciences, Gomal University, Dera Ismail Khan, Pakistan; ^5^ Department of General Practice, Henan Provincial Peoples Hospital, People's Hospital of Zhengzhou University, Zhengzhou, Henan, China; ^6^ School of Stomatology, Henan University, Kaifeng, Henan, China

**Keywords:** monkeypox (MPX), epidemiology, diagnosis, treatment, public health concerns

## Abstract

The world is currently dealing with a second viral outbreak, monkeypox, which has the potential to become an epidemic after the COVID-19 pandemic. People who reside in or close to forest might be exposed indirectly or at a low level, resulting in subclinical disease. However, the disease has lately emerged in shipped African wild mice in the United States. Smallpox can cause similar signs and symptoms to monkeypox, such as malaise, fever, flu-like signs, headache, distinctive rash, and back pain. Because Smallpox has been eliminated, similar symptoms in a monkeypox endemic zone should be treated cautiously. Monkeypox is transmitted to humans primarily via interaction with diseased animals. Infection through inoculation via interaction with skin or scratches and mucosal lesions on the animals is conceivable significantly once the skin barrier is disrupted by scratches, bites, or other disturbances or trauma. Even though it is clinically unclear from other pox-like infections, laboratory diagnosis is essential. There is no approved treatment for human monkeypox virus infection, however, smallpox vaccination can defend counter to the disease. Human sensitivity to monkeypox virus infection has grown after mass vaccination was discontinued in the 1980s. Infection may be prevented by reducing interaction with sick patients or animals and reducing respiratory exposure among people who are infected.

## 1 Introduction

The monkeypox virus (MPXV) causes monkeypox (MPX), an uncommon viral illness. MPXV is an Orthopoxvirus genus Poxviridae family member. Vaccinia, variola, and cowpox viruses are other members of this genus that may infect people (CDC, 2021a). There are signs similar to those of smallpox patients, but they are less severe. MPX is an infectious disease transmitted from animals to humans. Since the eradication of smallpox in 1980, MPX has become one of the most prevalent orthopoxviruses, especially in tropical forests. MPX is widespread throughout Central and West Africa (WHO, 2019). Fever, swelling lymph nodes, muscle aches, and fatigue are common symptoms. A rash with blisters and crusts then follows it. Although they typically occur one to two weeks after infection, MPX symptoms can appear up to 21 days after someone is exposed (Fenner et al., 1988a; Khodakevich et al., 1988; Di Giulio and Eckburg, 2004). There is an intense deep-seated, vesicular or pustular rash with a central spread 1-4 days after the prodrome. The scratches are highly defined and commonly umbilicate or become adherent, eventually resulting in scabs, and the rash can spread. Several current cases started with vaginal and perianal lesions but no particular temperature or other prodromal indications.

As a result, instances may be misdiagnosed as more frequent conditions like varicella-zoster or sexually transmitted. However, MPX could have a larger prevalence of sensitive people (McCollum and Damon, 2014; Durski et al., 2018; Petersen et al., 2019a). MPXV spreads through bushmeat, animal bites or scrapes, contaminated products, body fluids, or personal interaction with a septic individual. Numerous rodents in Africa have been infected by the virus (WHO, 2021). MPX disease may be diagnosed by extracting MPXV DNA from a patient’s blood sample and growing it in a viral culture (Moore and Zahra, 2021). The illness resembles chickenpox in appearance (McCollum and Damon, 2014). Vaccination against smallpox defends against infection (Yinka-Ogunleye et al., 2018). Currently, there is no established, safe therapy for MPXV illness. Antiviral drugs, vaccinia immune globulin (VIG), and smallpox vaccination can or have been utilized in the United States of America (USA) to control MPX infections. Cidofovir and brincidofovirin’s effectiveness in treating MPX is unknown. Studies in animals and *in vitro* have shown that both are effective against poxviruses (CDC, 2021b).

First human cases were documented in 1970 in the Democratic Republic of the Congo (DRC) (Bunge et al., 2022). In the West and Central Africa region, MPX still occurs infrequently despite the World Health Organization’s (WHO’s) 1980 declaration that Smallpox had been eradicated (WHO, 2016). A pet store that imported mice from Ghana in 2003 caused an epidemic in the United States (CDC, 2018; Yinka-Ogunleye et al., 2018). From October 2017 to February 2018, the recent MPX pandemic in Nigeria was catastrophic to the healthcare system (Fowotade et al., 2018). In the current MPX incident, a UK resident who arrived in Nigeria on April 20, 2022, visited Lagos and Delta State before departing Lagos on May 3, 2022, and landing in the UK on May 4, 2022, was involved (Muanya and Onyedika-Ugoeze, 2022). Several experts, including specialists, were unable to identify what caused the epidemic early on. In September 2017, the following MPX occurrence in Nigeria, the country regularly reported isolated viral cases from across all states, as per Nigeria Center for Disease Control (NCDC) (2022). 22 states reported 558 cases and eight deaths between September 2017 and April 30, 2022.There are 46 suspects, and fifteen confirmed cases from seven states had been registered with no recorded death (Nigeria Centre for Disease Control (NCDC), 2022). For its virulence, which is just next to the Variola virus, the source of Smallpox, with a 10% rate of death, MPXV is a potential biological warfare weapon (McCollum and Damon, 2014) like SARS-CoV-2 (Khattak et al., 2021a; Khattak et al., 2021b; Ullah et al., 2021; Ahmad et al., 2022; Khattak et al., 2022a).

Consequently, clinicians and the general population must be informed about its diagnosis, management, and control. This review will examine the present state of knowledge on human MPX, focusing on epidemiology features, diagnosis, prevention, clinical aspects, and therapy. Furthermore, the increasing number of MPX cases in non-endemic countries has made it a public health threat for other countries like Pakistan (Khattak et al., 2022b). The situation requires disease surveillance at a country level, and timely detection and notification of suspected cases are essential for effective preventive measures.

## 2 MPXV structure, genome and morphology

The morphology of MPXV shows that virions are ovoid or brick-shaped particles encased by geometrically corrugated lipoprotein outer membrane, sharing the same physical traits as other orthopoxviruses. The size estimates for MPXV have been confirmed to be 200 to 250 nm (Cho and Wenner, 1973). The outer membrane protects the membrane-bound and tightly packed core, double-stranded DNA genome, transcription factors, and enzymes. The core is characterized as a biconcave owing to an electron microscopy obsession artifact and has an adjacent body on either side (Jahrling et al., 2007; Odom et al., 2009).

**Figure 1:**
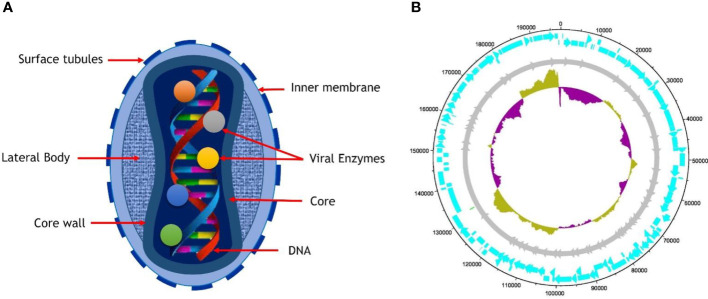
**(A)** Structure of mature virion of MPXV, **(B)** The complete genome of MPXV isolates SI2022 S7 (Genbank ID: ON838178.1) is 197652 bp long, with the length of the arrowhead representing genes (cyan) and the length of the arrow representing gene size. The dark yellow indicates that the GC content (33%) is above average, while the indigo color represents GC content below average.

The genomes of PVXM are composed of 197 kb of linear double-stranded DNA (Kugelman et al., 2014) that is intrinsically joined across both ends *via* the inverted terminal repeats (ITRs), consisting of tandem repeats hairpin loop as well as several open reading frames (ORF) and palindromic hairpins. Even though MPXV is a DNA virus, it spends its entire life cycle in the cytoplasm of infected cells. The MPXV genome encodes all proteins essential for viral DNA replication, transcription, virion assembly, and egress. Housekeeping function genes are preserved across Orthopoxvirus (OPVs) and found in the genome’s central area. In contrast, virus-host interaction genes are less conserved and located in the termini area (Cho and Wenner, 1973; Esposito and Knight, 1985; Takemura, 2001; Jahrling et al., 2007; Boyle and Traktman, 2009; Remichkova, 2010). Intracellular mature virus (IVM) and extracellular-enveloped virus (EEV) are the two kinds of infective virions formed by Vaccinia Virus (VACV) (and most likely MPXV). IVM is released when cells are lysed, but EEV is produced when cells come into contact with actin tails, leading to the virus’s quick long-distance spread within the host body. Whereas the traits listed above are unique to VACV, they are most likely shared through all OPVs (Remichkova, 2010). Cell-associated virions (CEVs) are produced when an inner encapsulated virus (IEV) travels to the edge of the cell and fuses with the plasma membrane, remaining connected to the cell’s surface. Cell-to-cell communication is essentially the responsibility of CEVs. IEV is produced when IMV is surrounded by a double membrane formed by an initial endosomal component (Jahrling et al., 2007) or the trans-Golgi network (TGN) (Schmelz et al., 1994). Aside from IEV exocytosis, another route for EEV production is IMV budding across the plasma membrane (Meiser et al., 2003). Virion morphogenesis in the prototype VACV may be erroneous, leading in non-infectious dense particles (DPs) (Meiser et al., 2003; Okeke et al., 2006), However, no reports of this for MPXV have yet been made. Additionally, contrast certain CPXV strains where IMVs are covered by A-type inclusions (ATI) (Shida et al., 1977; Okeke et al., 2006). MPXV does not generate ATIs or sequester IMVs within A-type inclusion (ATIs) due to the A-type inclusion body protein (ATIP) gene truncation (**Figure 1**) (Howard et al., 2010).

## 3 Epidemiology of MPX disease

### 3.1 Outbreak in Africa

Since humans were first infected with the virus through direct contact with diseased animals thousands of years ago, MPX has likely been present in Sub-Saharan Africa for thousands of years (Weinstein et al., 2005). There is currently no information about MPXV’s reservoir. Though, there is evidence that monkeys, like humans, are accidental hosts. The reservoir will likely be one or more mice or monkeys found in central Africa’s forest (Khodakevich et al., 1988). MPX was not known as a unique infection till 1970, when the eradication of Smallpox in Zaire (DRC) showed the persistence of a smallpox-like disease in rural regions. In the worldwide purging campaign, mass immunization in Central Africa appears to have resulted in a temporary decrease in the prevalence of human MPX. However, the disease has returned due to a lack of immunization in subsequent generations and a growing reliance on hunting animals in conflict-torn regions (Weinstein et al., 2005). A total of 47 human cases of MPX have been reported in Sub-Saharan Africa from 1970 to 1979, with 38 of these occurring in the DRC and the remaining taking place in Cameroon, the Central African Republic, Gabon, Cote d’Ivoire, Liberia, Nigeria and Sierra Leone (Breman et al., 1980; WHO, 1980). The DRC cases all occurred near tropical rainforests and appeared connected to animal interaction. Seven of the 47 diseases documented were lethal. The secondary spread was shown to be the leading prospective source of contagion in four cases, with the second occurrence proportions of 7.5% between nearby family members living in the same house and 3.3% between all exposed interactions. Many cases have been recorded in the DRC since 1980 (Weinstein et al., 2005).

From 1981 to 1986, the WHO conducted a comprehensive investigation in the DRC to determine whether MPX may arise from central Africa and fill the void left by Smallpox. From 1970 to 1986, 338 of the 404 recorded African instances occurred (Jezek and Fenner, 1988). In 245 of the 338 cases, an animal source of infection was suggested, and secondary spread from a human source was proposed in the remaining 93 cases. Most victims were children, with an average age of 4.4 years. These rises in the secondary transfer rate (3 times the 9% rate for patients in the 1970s) and demography were assumed to suggest a decline in immunity once vaccination was discontinued. Only four generations of human-to-human spread were seen in the largest recorded disease chain, indicating that MPXV had a limited capacity to cause epidemics (Jezek et al., 1986). During this period, serological investigations of vaccine-naive newborns revealed that 12–15% of the children, had antibodies to MPVX. However, the majority had no history of the associated illness, showing that asymptomatic transmission also existed (Jezek and Fenner, 1988). Since the WHO monitoring program stopped in 1986, few medical research works have reported the recent incidence of human MPX. Individual 13 cases were documented in the literature between 1986 and 1992, and none were recorded between 1993 and 1995 (Heymann et al., 1998).

However, in the Kasai-Oriental region of the DRC, over 500 probable MPX cases have been identified between 1996 and 1997 (Heymann et al., 1998; Hutin et al., 2001). Even just a very few of these cases were laboratory validated. The percentage of secondary cases was substantially more significant than the previous WHO research findings (78%). The death rate was markedly lower (1-5%), showing that many were varicella cases. There were no further confirmations of suspected MPX cases till 2001, when 31 people with MPX were found in 7 distinct disease groups in the Equateur Province of DRC.

In the DRC, healthcare contractors monitor passive disease despite governmental unrest and inadequate resources. Their results show that MPX is more frequent than in previous studies (Kebela, 2004). The DRC Ministry of Health received reports of 1265 instances between January 1, 1998, and December 31, 2002, with specimens collected in 215. MPXV was the cause of 88 of the 215 cases, according to PCR and viral culture. Patient ages varied from 10 months to 38 years for laboratory-confirmed cases, with a mean age of 16.5 and 15.5 years. Patients made up 73.2% of the population who were over 25, while 26% were children under 10 (Kebela, 2004).

### 3.2 The outbreak in the US

During the summer of 2003, a wave of disease cases associated with MPXV were confirmed in the midwestern United States (Reed et al., 2004). MPXV was discovered for the first time in the Western World. Thirty seven of 72 human cases in an outbreak were confirmed by laboratory tests (Control, C.f.D. and Prevention, 2003; Control, C.f.D. and Prevention, 2003a; Sejvar et al., 2004). Because most infected people fell ill after associating with dogs, prairie dogs (Cynomys species) kept with rodents transported from Ghana in western Africa were assumed to be the primary source of the pandemic (Sejvar et al., 2004). Ignoring that viral infection seemed to happen *via* direct interaction with an infected dog, two patients cared for their ill children, and individual contamination could not be excluded entirely (Reed et al., 2004).

Even though no symptoms or clinical signs were observed in a new research work of 81 health care employees who contacted three people with MPX infection, one asymptomatic health care employee had laboratory proof orthopoxvirus contagion, which could be attributed to also current infection or smallpox vaccination (Fleischauer et al., 2005). There was a mild, self-limiting feverish rash disease among most African patients who contracted the disease during the US outbreak. 18 of the 69 people whose data was available were from the hospital. However, several were just admitted for isolation measures (Control, C.f.D. and Prevention, 2003a). Two patients, both youngsters, were suffering from severe clinical illness (Missouri, Ohio, and Wisconsin, 2003; Anderson et al., 2003; Control, C.f.D. and Prevention, 2003a). The first infant got severe encephalitis and needed 14 days in an intensive care unit (Missouri, Ohio, and Wisconsin, 2003; Sejvar et al., 2004). Encephalitis is a relatively unusual consequence of MPX, with only one prior report (Ježek et al., 1987; Jezek and Fenner, 1988). The second youngster was admitted to the hospital with tonsillar lymphadenopathy and agonizing symptoms of cervical and oropharyngeal pox (Anderson et al., 2003). Both kids were cured, and no one resulted in the epidemic.

Surprisingly, just one patient (a toddler) developed a broad rash comparable to earlier African patients. Many others had limited lesions on their fingers and hands due to exposure to an infected animal, which could be because inoculating a strain of MPXV through prairie dog bites results in significantly milder disease than inhaling the same strain, which is also less lethal as isolates from the Congo basin (Chen et al., 2005).

## 4 Human MPX

**Figure 2:**
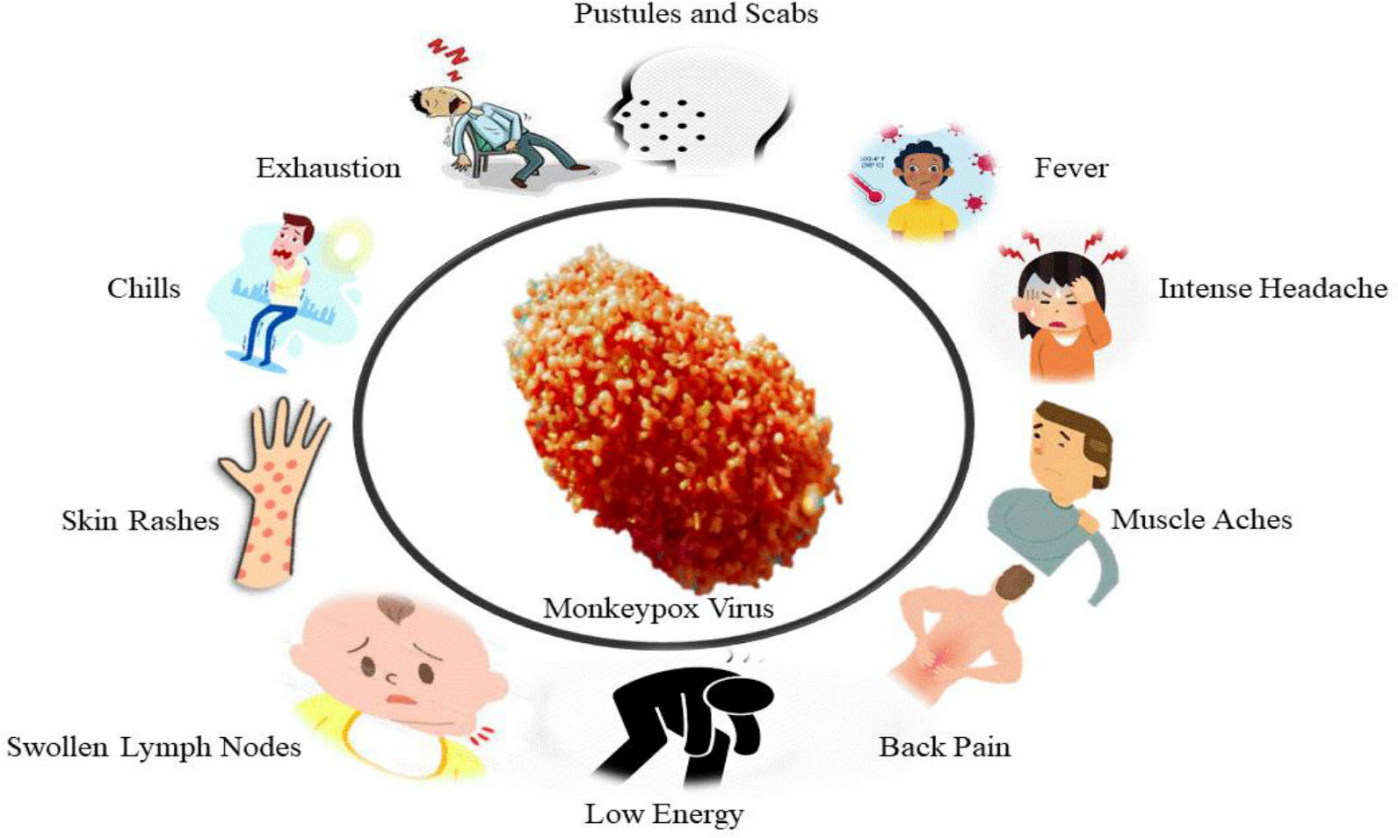
The sign and symptoms of MPX Disease.

In 1970, a 9-month-old Zaire kid was diagnosed with MPX as a human disease (50, 51). Till now, utmost human MPX medical information originated from epidemic research in Western and Central Africa. Humans are believed to contract the virus through contact with diseased animals’ body fluids or lesions. Significant breathing droplets might be transferred in extended face-to-face contact, although less effective than smallpox (Fenner et al., 1988b). Human MPX clinical characteristics are quite similar to ordinary Smallpox (Breman et al., 1980). It usually takes 10 to 14 days of incubation for patients to develop a rash after experiencing prodromal symptoms such as fever, malaise, and enlarged lymph nodes (**Figure 2**) (Jezek and Fenner, 1988; Di Giulio and Eckburg, 2004). Chills, sweating, backache, headache, sore throat, shortness of breath, and cough are also indications and symptoms of MPX. MPX is characterized by lymphadenopathy, Smallpox is not a common disease, being reported in 90% of unvaccinated individuals. Swelling of lymph nodes can happen in the neck, submandibular or inguinal regions (Fenner et al., 1988a). Before the typical maculopapular rash occurs, the prodromal stage usually lasts 1–3 days. The patient is contagious in the first week of the rash and should be quarantined until all scabs have been removed and throat swab PCR results are negative. Its skin lesions are 0.5–1 cm in diameter and progress from macules to papules, vesicles, and pustules, followed by scabbing, desquamation, and umbilication over 2-4 weeks (Fenner et al., 1988a). A circular spread of the rash may occur on the palms and soles of the feet, even though it appears first on the neck. Mouth and tongue lesions, as well as vaginal lesions, are also possible.

In contrast to skin wounds and lesions, individuals infected with MPXV might develop extra-cutaneous symptoms such as Pneumonitis (12% of cases), encephalitis (1%), secondary skin and soft tissue infection (19%), and ocular complications (4-5%) (Di Giulio and Eckburg, 2004).

## 5 Transmission

**Figure 3:**
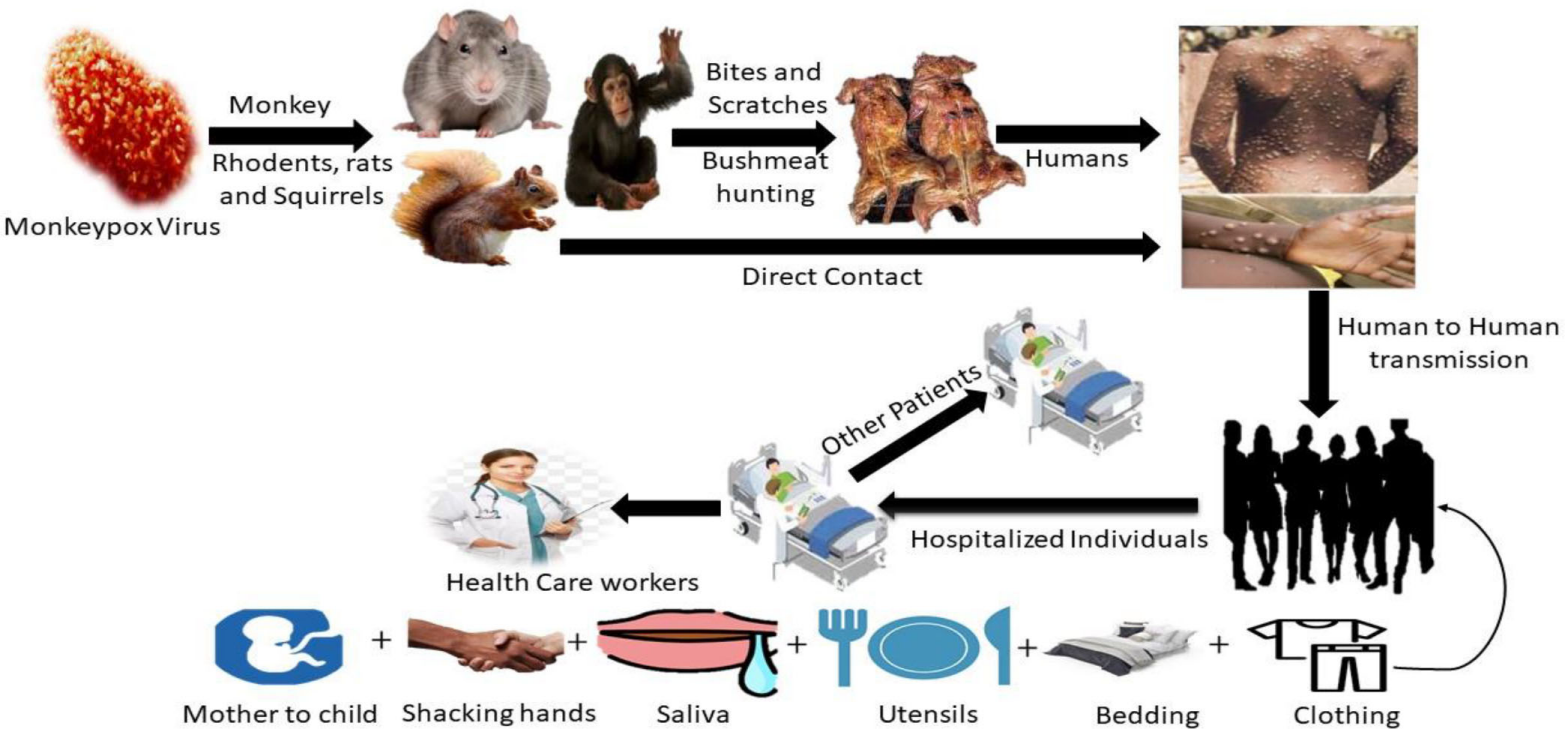
The primary host and transmission route of MPX, eating infected animals as a food source, is the major cause of animal-to-human virus transmission. Healthy people can contract the virus by intimately contacting someone who is infected.

Contamination of animals with blood or bodily fluids; direct contact with blood or bodily fluids. Ingestion of insufficiently prepared meat from infected animals, inoculation from infected animals’ mucocutaneous sores, particularly once the skin barrier is ruptured due to bites, scratching, or other disturbances, or inoculation from mucocutaneous lesions on diseased faunae are methods for the virus to reach people (**Figure 3**) (Sutcliffe et al., 2012; Pal et al., 2017). Interacting diseased monkeys, rats, rabbits, Gambian giant squirrels, Porcupines, dormice, prairie dogs, and gazelles has been identified as a possible transmission mode (Pal et al., 2017). Direct physical interaction is the most common source of infection in MPX outbreaks, in small groups where people hunt and assemble. Even though no specific species have been found, rodents are studied as a reservoir. When large respiratory droplets come into touch with one another, they can transfer infection from person to person. Disease with MPX has the potential to spread through the placenta (Pal et al., 2017).

MPX infection begins with a cutaneous or respiratory epithelial infection in affected animals or people. A primary viremia results in systemic infection, followed by secondary viremia, which causes epithelia infection and ulceration in the skin and mucosa. Through oropharyngeal secretions, viruses replicate on mucosal surfaces and spread to close contacts. Even though MPXV is capable of evading host immune responses, the density of viral particles in oropharyngeal secretions, proximity and duration of contact, and viral survival affect virus transmission (Sutcliffe et al., 2012).

## 6 Reservoirs and hosts

It is believed that rodents are the primary reservoir in Africa; a study showed that some forest-dwelling rodents can be infected by Orthopoxvirus (including MPX) (**Figure 3**) (Khodakevich et al., 1986; Huhn et al., 2005). Serological studies show that various species, such as squirrels, nonhuman primates, and rats, are infected with MPV in the wild. Numerous observational studies in the DRC have revealed squirrels (particularly Funisciurus anerythrus) residing in farming regions as potential carriers of viruses to people living in nearby towns (Khodakevich et al., 1986). Funisciurus spp. squirrels, compared to Heliosciurus spp. squirrels (15%) and primates (8%), had a higher rate of seropositivity for MPV (24%). In February 1997, a second seroprevalence analysis conducted as a segment of the inquiry into the DRC epidemic revealed significantly greater positive proportions in these squirrels (39 to 50% in Funisciurus spp. and 50% in Heliosciurus spp. squirrels) (Hutin et al., 2001). Furthermore, this study identified serological confirmation of MPV infection in 16% of the Gambian giant rats. After contacting an ill prairie dog, a rabbit contagion (family Leporidae) at a veterinary clinic established the virus’s transmissibility between North American animal species. Less is known regarding MPV and HIV co-infection (Di Giulio and Eckburg, 2004).

## 7 Diagnosis

The clinical manifestations of MPX are not the same as that of Smallpox and chickenpox. A correct assessment is crucial for treating illnesses or early detection of a potential bioterrorism occurrence. Although infections caused by parapoxviruses, e.g., bovine stomatitis and orf, can create little skin wounds and lesions identical to those recognized in the US MPX epidemic, electron microscopy clearly distinguishes them from orthopoxviruses. As FDA-approved antiviral treatment for MPX, once the illness pathogen has been recognized, the appropriate public health prevention methods are quarantine and fast ring vaccination. Due to the apparent efficiency with which direct touch and aerosol particles can transmit disease, aseptic conditions should be maintained by using respiratory measures when handling samples such as a scab. Although clinical features help differentiate poxvirus illnesses from many further causes of vesiculopustular rashes, laboratory diagnosis is essential for precise identification.

Laboratories’ investigative procedures for MPX contain IgM, IgG, Enzyme-linked immunoassay (ELISA), electron microscopy, Polymerase Chain reaction (PCR), Virus Isolation, Immuno-fluorescent antibody test, and histopathologic examination. Regrettably, most of these approaches are ambiguous and cannot distinguish MPXV disease from those other poxvirus diseases. For example, lesions caused by the MPXV histologically resemble those caused by the varicella-zoster, cowpox, variola, and herpes simplex viruses, with massive fibroblast degeneration, cutaneous edema, acute inflammation, and severe spongiosis (Bayer-Garner, 2005). However, immunohistochemically examination containing monoclonal and polyclonal antibodies in contradiction of all orthopoxviruses can help distinguish a herpes virus infection from a pox virus disease. In the past, electron microscopy was frequently used to aid viral diagnosis (Gentile and Gelderblom, 2005).

A laboratory with a biosafety level-3 should conduct PCR or real-time PCR (Fowotade et al., 2018). In clinical and veterinary specimens as well as in cell cultures with MPXV infection, MPXV DNA is routinely detected using real-time PCR using conserved regions of the extracellular envelope protein gene (B6R) (Li et al., 2006), DNA polymerase gene, E9L (Yinka-Ogunleye et al., 2019). Rpo18, a DNA-dependent RNA polymerase subunit, and the F3L gene (Reynolds et al., 2010; Orba et al., 2015). PCR-amplified genes or gene fragments are also examined by restriction-length fragment polymorphism (RFLP) to detect MPXV DNA (Meyer et al., 1994; Kulesh et al., 2004). However, RFLP takes time and requires viral culture. In clinical settings where speed, sensitivity, and specificity are critical, RFLP of PCR products may not be the best approach because it also requires enzyme digestion and gel electrophoresis. MPXVs and other OPVs remain best characterised by whole-genome sequencing using next-generation sequencing (NGS) methods (Radonić et al., 2014; Cohen-Gihon et al., 2020). Despite its advantages, downstream sequencing technology is expensive and requires a great deal of computer power.

In resource-constrained areas like Sub-Saharan Africa, NGS may not be the most efficient characterization technique. While real-time PCR remains the preferred approach for routine MPXV diagnosis, it must be augmented *via* field genome sequencing technology, including Oxford NanoporeMinION, to provide epidemiological interventions based on evidence-based viral genome data in real time. In resource-constrained parts of West Africa, during the outbreak of Ebola, MinION field sequencing was used effectively for genomic surveillance (Quick et al., 2016).

The MPXV incubation period is 4-21 days, according to clinical diagnosis, and a prodromal disease frequently accompanies it with various symptoms such as lymph node enlargement, fever, myalgia, headache, back pain, strong asthenia, malaise, pharyngitis, and drenching sweats. In the prodromal phase, a vesiculopustular rash appears between 1-10 days of development and spreads throughout the body during the exanthema phase. In MPXV patients, the lesions appear to be monomorphic, pea-sized, and hard, similar to smallpox lesions. Smallpox can be distinguished from MPXV by the crop-like form of the lesion and the absence of vigorous centrifugal spread. MPXV can also be distinguished from smallpox by lymphadenopathy (McCollum and Damon, 2014; Sklenovska and Van Ranst, 2018; Kabuga and El Zowalaty, 2019). It is imperative to make early identification of suspected MPX cases, even without laboratory confirmation. In a cohort of 645 patients, the clinical definition of MPX had high sensitivity (93-98%). However, the specificity is low (9-26%) (Osadebe et al., 2017; Beer and Rao, 2019).

The detection of viral antigens is accomplished by immunohistochemistry, and the detection of IgG and IgM antibodies is accomplished by ELISA. Immunochemistry analysis can be performed using polyclonal or monoclonal antibodies in contradiction of entirely OPVs to distinguish amid poxvirus and herpes virus infection. Antiviral antibody levels, as well as T-cell responses, have been shown to rise around the time of illness onset. IgM and IgG, however, can be detected in serum between five and eight days after the rash begins. An individual without a history of rash or severe illness who tests positive for IgM and IgG antibodies, an indirect MPXV diagnosis may occur. However, none of these strategies is unique to MPX (McCollum and Damon, 2014; Nasir et al., 2018; Petersen et al., 2019b; Sadeuh-Mba et al., 2019). In addition to other OPV species, IgM can be utilized to diagnose MPX infection in people who have previously been vaccinated against smallpox (Weaver and Isaacs, 2008). The IgM capture ELISA indicates recent contact to OPV (possibly MPXV in endemic areas), while the IgG capture ELISA indicates prior exposure to OPV (Karem et al., 2005; MacNeil et al., 2011). Thus, OPV antibodies in a sample indicate recent exposure by individuals who have previously received the vaccine or susceptible to spontaneous infection. In MPX-endemic areas, IgM is found in individuals who are already immunized versus smallpox.

Similarly, if accessible, electron microscopy can be used as a laboratory for identifying poxvirus contagions. It might be one of the early signs of a rash disease. Under electron microscopy, typical poxvirus virions with the characteristic morphology would be predictable to be detected. For example, during a current MPX epidemic in the United States, electron imaging revealed keratinocytes containing a substantial proportion of mature virions along with immature virions in the stage of synthesis (also called “viral factories”) inside the cytoplasm (Bayer-Garner, 2005). This approach, though, cannot distinguish between orthopoxvirus species. Virus isolation and classification through different PCR methods, whether restriction fragment length polymorphism testing or amplicon sequencing, is frequently regarded as conclusive for MPXV identification (Murray et al., 2006). Furthermore, the accessibility of real-time PCR that employs panorthopoxvirus or MPXV-specific targets has expanded (Kulesh et al., 2004; Olson et al., 2004). Another quick approach for detecting orthopoxviruses has been developed; a DNA oligonucleotide microarray containing the TNF receptor gene crmB (Lapa et al., 2002).

Regardless of the epidemic, doctors should evaluate MPX in patients with a novel onset pyretic temperature and rashes if lymphadenopathy is evident. The rashes generally start on the lips and spread to the cheeks and extremities in a centrifugal pattern (including the palms and soles). PCR analysis of skin lesions or fluids yields a precise diagnosis. Such diagnostics are available exclusively at national public health labs; no commonly accessible screening is available (Centers for Disease Control and Prevention, 2022; Adalja and Inglesby, 2022).

## 8 Treatment

MPX infection is currently without a clinically validated therapy. The therapy, like with most viral diseases, is symptom control. There are, though, preventative methods that can assist avoid an epidemic.

The septic person must be isolated, keep covered lesions and wounds, and wear a mask even more than probable till all lesion crusts break off and a new skin layer form. In extreme circumstances, for exploratory usage, medicines with efficacy against orthopoxviruses in animal trials and severe vaccinia vaccination sequelae may be evaluated. There is an approved treatment for smallpox known as brincidofovir, tecovirimat, and vaccinia immunoglobulin, as well as an inhibitor of intracellular viral release known as tecovirimat and which has shown efficacy against MPX in animals (Reddy, 2018; Bunge et al., 2022; WHO, 2022c).

Following exposure, measurement of temperature and symptoms should be done twice daily for 21 days since that is the recognized maximum incubation period for MPX. Because infectiousness co-occurs as illness starts, close contacts do not need to be separated when asymptomatic. Vaccination with the Ankara vaccine after vaccinia exposure (live, non-replicating smallpox vaccine) is advised in certain situations. Interaction among wounded skin or mucous membranes with the bodily fluids, respiratory droplets, or scabs of an infected patient is thought to be a “high risk” contact that necessitates post-exposure vaccination as soon as possible. After close connection with an MPX case, it is recommended that vaccination take place within four days after first contact with the virus, but it is possible to give the vaccination up to 14 days after that (Control, C.f.D. and Prevention, 2003b).

A vaccination containing a replication fault, the Ankara vaccine is a two-dose vaccine administered four weeks separately and has a better profile than first- and second-generation smallpox vaccination. Ankara injection, contrasting live vaccinia virus training, does not cause skin lesions or provide a danger of extensive or local transmission (McCollum and Damon, 2014). The results of medical studies confirmed that altered “vaccinia Ankara” is safe and increases antibody production in people with early or weakened immune systems, both of which are limitations of delivering live vaccinia to individuals (Petersen et al., 2019).

**Figure 4:**
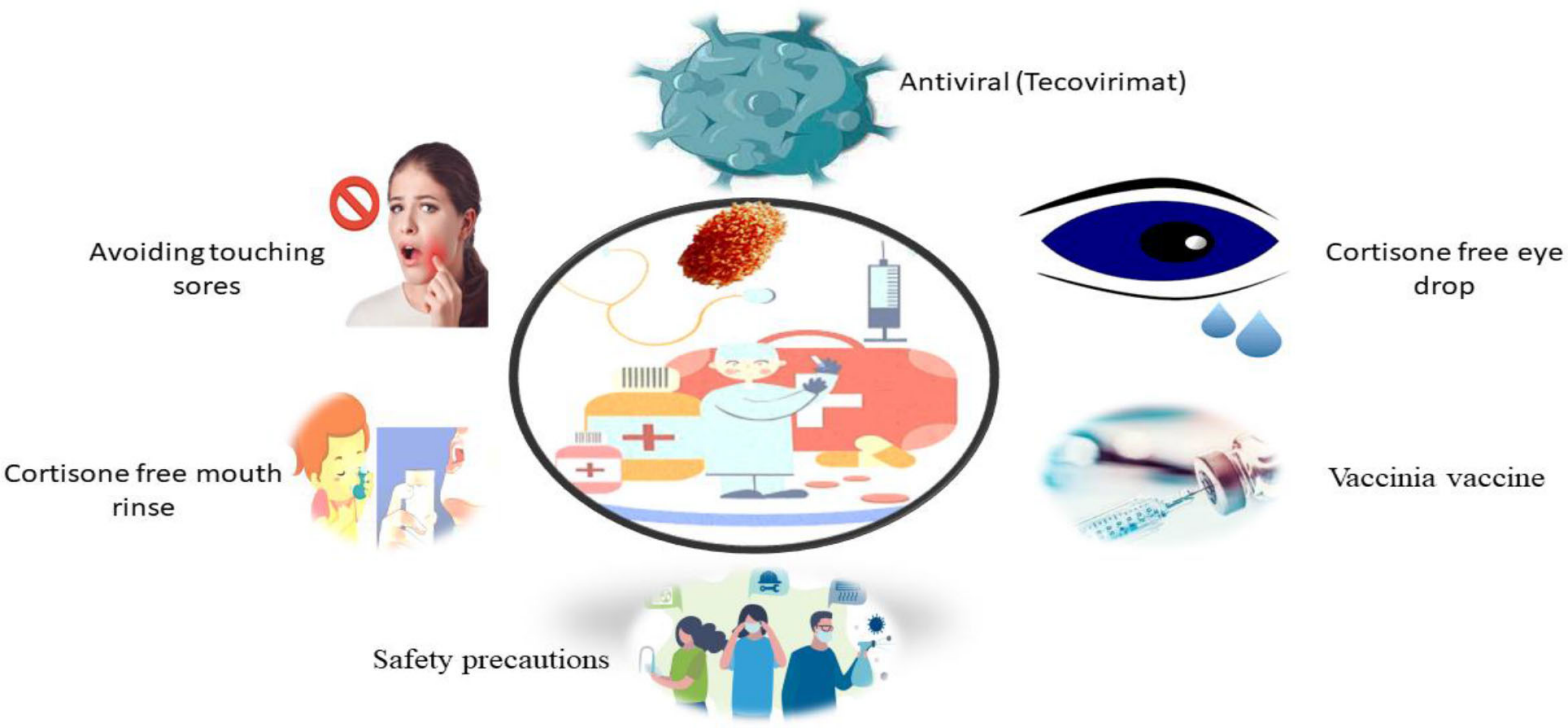
Possible treatment of MPX Virus Disease.

More detailed data and feasibility studies are needed to identify preventive MPX immunization’s potential benefits and drawbacks in endemic areas. The availability of therapeutic care diagnosis and facilities limits the ability to create knowledgeable judgments on managing this deserted tropical ailment (Reynolds et al., 2017; Petersen et al., 2019). While, MPX does not have any specific treatment, such as brincidofovir, tecovirimat, vaccinia immunoglobulin, and tecovirimat, which has shown efficacy against MPX in animals (**Figure 4**) (Costello et al., 2022; Moore and Zahra, 2022). CMX-001, ST-246, and Cidofovir are other promising antivirals (McCollum and Damon, 2014). The US Food and Drug Organization has authorized the usage of the latter two drugs in smallpox treatment (FDA). Such therapies would be earmarked for severe cases or immunocompromised individuals and provided through a public health agency or the CDC (Centers for Disease Control and Prevention, 2022; Adalja and Inglesby, 2022).

## 9 Control and prevention

**Figure 5:**
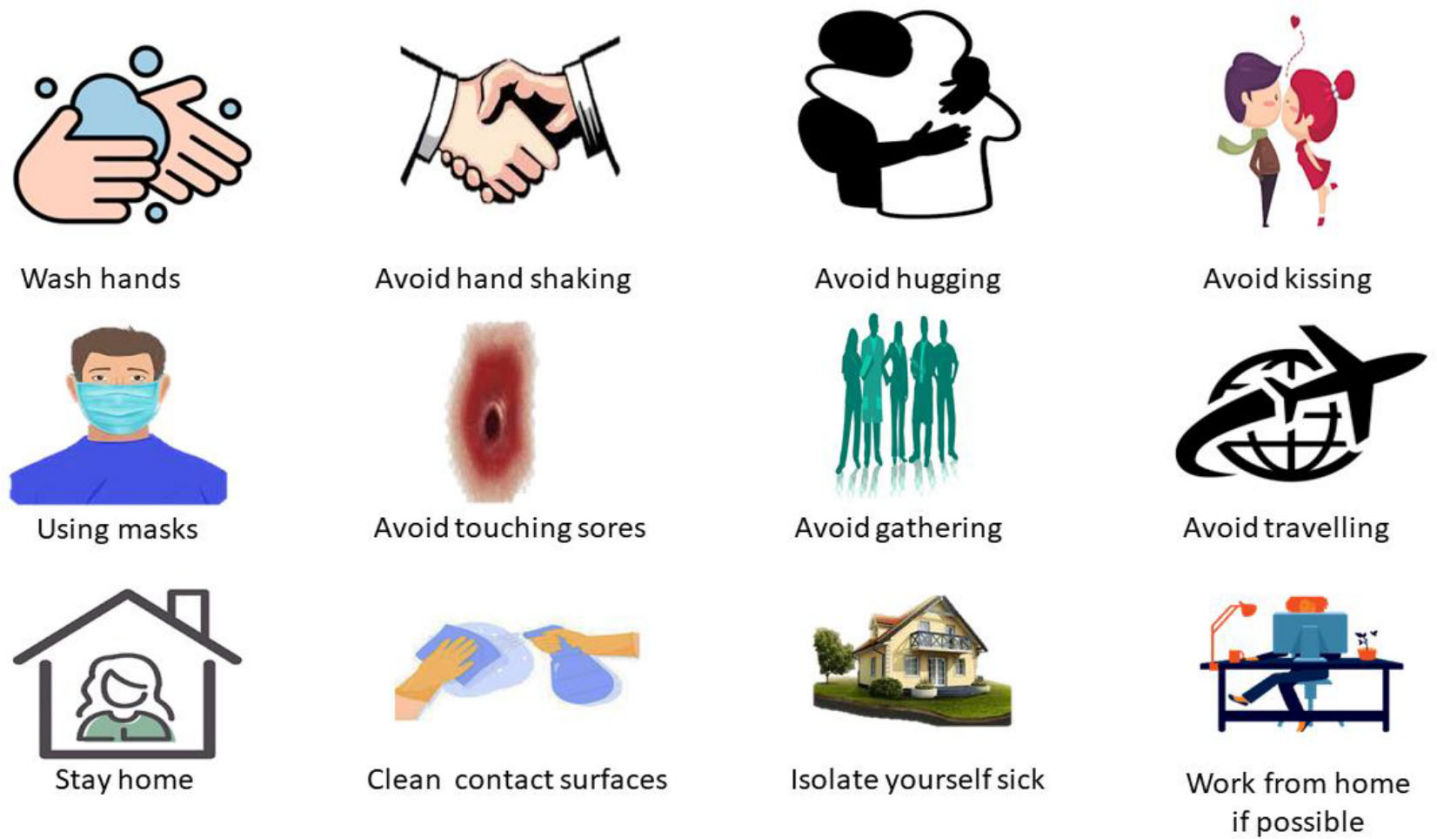
Preventive measures to control the MPX.

Enhanced infection control procedures, like regular screening and isolation of new infections animals, would surely benefit epidemic control (**Figure 5**). Proper hygiene habits are essential to prevent the virus from spreading on fomites and becoming a source of new infections. Vaccination with the vaccinia virus may be an alternative for animal protection. Because diseases have been observed in Asian monkeys mixing with African primates, these species must be maintained apart (Pal et al., 2017; Quiner et al., 2017). To avoid spreading the disease, someone infected with it should minimize interaction with animals, notably mice and animal primates (Quiner et al., 2017).

Take the following actions to prevent infection with the MPXV:

1). Avoid any animals that may be infected with the virus (animals that are sick or dead in areas where MPX occurs).
2). Avoided things that interacted with infected animals and humans.
3). While caring for the infected, use personal protective equipment, including gowns, masks, respirators, gloves, shoe covers, goggles, eye protectors, and face shields.
4). Isolate sick individuals from people who may have been infected.
5). When interacting with sick humans or animals, ensure proper hand sanitation by cleaning hands with soap or sanitizer.

Smallpox vaccinations are applicable against MPX. The FDA has approved ACAM2000, a newer-generation smallpox vaccine, for treating MPX (Hatch et al., 2013; Koenig et al., 2022; Rao et al., 2022), the older generation of ACAM2000 can also be used off-label for the same reason. Vaccinating close contacts has proven to be effective in controlling transmission of previous epidemics. As soon as feasible after probable exposure, prophylactic vaccination administration can abort or dramatically reduce infection. If smallpox vaccination is unavailable, vaccinia immune globulin is possibly an alternate post-exposure prophylactic negotiator (Centers for Disease Control and Prevention, 2022; Adalja and Inglesby, 2022). While in an epidemic, MPX viral transmission can be prevented by isolating affected animals (at least for six weeks from the day of the contract) and tracking their interaction. The regions where these animals were housed should be carefully decontaminated. Specific directions from the healthcare-related authority or the CDC Website must be followed.

## 10 Reasons for the re-emergence of MPX disease

Several interconnected causes have contributed to the recent comeback of infectious illnesses. Global travel and business are opening up the world to more people. The deepening of economic, political, and cultural ties emphasizes relationships between humans and other animals. These interact and engage in both accidental and intentional microbial agent transmissions. The United Kingdom reported three outbreaks of MPX between May 25 and June 15, 2021 (Reynolds et al., 2019; Okyay et al., 2022; WHO, 2022a). On May 8, 2021, a first case from Nigeria was identified. This comment might be utilized to highlight the importance of global travel assistance in virus transmission. Although inter-human transmission is limited, particularly in healthcare and the home, it causes epidemics.

Nevertheless, studies indicate that human infections will not emerge without recurring zoonotic invasions. Expanding animal-human connections increases the likelihood of infection from animals to humans (Reynolds et al., 2019; Okyay et al., 2022; WHO, 2022a). As a result, one of the essential goals in the fight against this disease is limiting virus transmission from animals to humans. Preventive methods include avoiding contact with diseased animals and ill or dead animals located in infested regions, in the case of handling materials that have come into contact with sick animals, including furniture, and segregating infected patients from others who may be diseased. Hands should be washed with soap and water after touching infected animals or humans, or utilize an alcohol-based hand sanitizer. To prevent MPX, it is important to raise awareness of risk factors and educate individuals about how to decrease their exposure to it (Sklenovska and Van Ranst, 2018).

It should be noted that the usage of smallpox vaccination may offset the virus’s return. MPX, initially found in the DRC in 1970, is most likely the result of a lack of immunization following the elimination of smallpox. Concerns about the eradication of smallpox through vaccination due to the presence of the disease in the exact geographic location with varying epidemiological aspects and field outcomes (Sarwar et al., 2022). The interruption of vaccine administration may increase the number of susceptible persons. Another issue that may cause the disease to reemerge is a failure to offer vaccination to susceptible persons in places where Human immunodeficiency virus (HIV) infection is widespread. Evaluating the immunization program, identifying susceptible individuals, and assuring vaccination are vital. Though there have been isolated incidences of MPX outside African countries, insufficient work has been devoted to producing a specialized vaccine to prevent the infection (Heymann et al., 1998) concerning the recent return of infectious illnesses during an epidemic.

Invasive procedures (such as angiography labs) and therapeutic medications used in the prevention of thromboembolic events have become more widely used, as the global population grows, all lead to the average life expectancy being prolonged as technology advances. The growing global population, along with rising average life expectancy, predicts a rise in the population of sensitive person. Vaccination discontinuation, expanding population, longer life expectancy, and more worldwide relations resulting from more straightforward transportation are prospects for the re-emergence of MPX illness (Okyay et al., 2022).

## 11 Public health strategy

Health experts are currently monitoring all individuals to determine when the isolation period will end (i.e., when all scabs have fallen off and fresh, healed skin appears). The CDC made available tecovirimat, an antiviral licensed for Smallpox but with antiorthopoxvirus action, through extended access from the strategic national stockpile (Merchlinsky et al., 2019). The CDC also made vaccination, PEP accessible to contacts who had high-risk exposures (for example, it would be dangerous to contact a patient’s skin or mucous membranes unprotected or be exposed to their bodily fluids (i.e., being within 6 feet of an unmasked patient for 3 hours with no protective measure). In low or unknown-risk circumstances (for example, new health care providers), PEP is not recommended. PEP with ACAM2000 or JYNNEOS vaccinations is administered to appropriate intermediate and high-risk contacts. The interaction research is in progress; of the 13 patients who have recognized interactions, 56 are considered high risk, 117 are regarded as intermediate risk, and 235 are considered low or unknown risk. For 21 days following the last encounter, contacts should be observed for symptoms and indications of MPX. According to DNA sequencing results of the virus obtained from the Massachusetts patient, it resembles other genomes described in this European outbreak (Next strain/MPX) (Genomic epidemiology of monkeypox virus, 2022). They are linked to the 2017–2018 MPX pandemic in Nigeria. According to early statistics, as of June 6, around 800 cases of MPX have been recorded in this epidemic from 28 countries, such as the United States (Monkeypox Outbreak — Nine States, 2022).

## 12 Concerns regarding the ongoing epidemic

MPX has been documented in North America, the US, and Europe. These cases exist outside the endemic area of the virus, and are transmitted by person-to-person. Several seemingly unrelated groups have formed based on the fact that most of them come from a prevalent country. Most cases are reported in sexually transmitted infection (STI) hospitals as well as in men who have had sex with men (MSM) (WHO, 2022b). In this population, the virus may or may not transmit sexually rather than *via* skin-to-skin contact and droplet respiratory spread. In MSM clusters, the latter has been identified as a transmission mechanism for meningococcus. The most critical challenge is to determine how the epidemic started. Compared to the previous MPX epidemic outside of Africa, why is this epidemic so much more widespread and significant? There are no genetic variants that are believed to promote transmissibility, according to preliminary genetic research (Isidro et al., 2022). Case-control training and quick case examinations are now underway and are essential for understanding this problem.

In the meantime, efforts will be focused on detecting cases, diagnosing them early, tracking contracts, isolating patients, and vaccinating them after exposure. If MPX epidemics continue, medical professionals, outpatient treatment providers, critical care clinicians, dermatologists, and clinics dealing with STIs could identify additional cases.

## 13 Conclusion

It is likely that MPX has existed in Sub-Saharan Africa since humans first came into contact with diseased animals thousands of years ago. Humans can exhibit signs and symptoms that are disturbingly similar to chickenpox, Smallpox, or other vesiculopustular rashes. An outbreak requires precise and prompt laboratory identification.

MPX cases in Africa are closely associated with Smallpox cases and the population is developing antibodies deficiency as a result of the discontinuation of routine Smallpox vaccination has raised concerns that MPXV may be used as a bioweapon. As a result of these factors, MPXV, along with the variola virus and many other poxviruses, is on the NIH’s highest danger list. The CDC has categorized it as a “select agent.” Human travel is prevalent today, providing risk for the spread of MPX, and animals carried across borders represent an immediate danger of disease spread. Because biological warfare potential cannot be ruled out, enhanced knowledge of MPXV and related virus may improve emergency management.

## Author contributions

All authors listed have made a substantial, direct, and intellectual contribution to the work and approved it for publication.
